# Supplementary material for: End Sequence Analysis Toolkit (ESAT) expands the extractable information from single-cell RNA-seq data
Source: Genome Res. 2016 Oct;26(10):1397–410. doi: 10.1101/gr.207902.116 (PMC5052061; doi:10.1101/gr.207902.116)
Supplement: Supplemental Material [file supp_gr.207902.116_Supp_Materials.tar.gz › ESAT-esat_v0.1/doc/html/classumms_1_1core_1_1model_1_1score_1_1_ratio_score.html]

ESAT: umms.core.model.score.RatioScore Class Reference


ESAT


- Main Page
- Related Pages
- Packages
- Classes
- Files

- Class List
- Class Index
- Class Hierarchy
- Class Members

All Classes Namespaces Files Functions Variables Enumerator Pages

- umms
- core
- model
- score
- RatioScore

Classes |
Public Member Functions |
Static Public Attributes |
List of all members

umms.core.model.score.RatioScore Class Reference

Inheritance diagram for umms.core.model.score.RatioScore:

[legend]

Collaboration diagram for umms.core.model.score.RatioScore:

[legend]

|  |  |
| --- | --- |
| Classes | |
| class | **Processor** |
|  | |
| class | **RatioScoreIterator** |
|  | |

|  |  |
| --- | --- |
| Public Member Functions | |
|  | RatioScore (Annotation a) |
|  | |
|  | RatioScore (Annotation numerator, Annotation denominator, double nTotal, double dTotal) |
|  | |
|  | RatioScore (Annotation numerator, Annotation denominator, double nTotal, double dTotal, double nRegionTotal, double dRegionTotal) |
|  | |
|  | RatioScore (CountScore numerator, CountScore denominator) |
|  | |
|  | RatioScore (AnnotationCollection<?extends Annotation > numerator, AnnotationCollection<?extends Annotation > denominator, Annotation annotation) |
|  | |
|  | RatioScore (AnnotationCollection<?extends Annotation > numerator, AnnotationCollection<?extends Annotation > denominator, Annotation annotation, double numeratorRegionTotal, double denominatorRegionTotal) |
|  | |
| void | setNumeratorCount (double d) |
|  | |
| void | setDenominatorCount (double d) |
|  | |
| void | setNumeratorTotal (double d) |
|  | |
| void | setDenominatorTotal (double d) |
|  | |
| void | setNumeratorRegionTotal (double d) |
|  | |
| void | setDenominatorRegionTotal (double d) |
|  | |
| double | getNumeratorCount () |
|  | |
| double | getDenominatorCount () |
|  | |
| double | getNumeratorTotal () |
|  | |
| double | getDenominatorTotal () |
|  | |
| double | getNumeratorRegionTotal () |
|  | |
| double | getDenominatorRegionTotal () |
|  | |
| double | getNumeratorRPKM () |
|  | |
| double | getDenominatorRPKM () |
|  | |
| double | getRatio () |
|  | |
| double | getScore () |
|  | |
| double | getEnrichmentBinomialScore () |
|  | |
| double | getEnrichmentBinomialPValue () |
|  | |
| double | getDepletionBinomialScore () |
|  | |
| double | getDepletionBinomialPValue () |
|  | |
| double | getLog2Ratio () |
|  | |
| double | getRegionRatio () |
|  | |
| double | getLog2RegionRatio () |
|  | |
| double | getRegionNumeratorRPKM () |
|  | |
| double | getRegionDenominatorRPKM () |
|  | |
| String | toString () |
|  | |
| Public Member Functions inherited from umms.core.model.score.CountScore | |
|  | CountScore (Annotation a) |
|  | |
|  | CountScore (CountScore other) |
|  | |
|  | CountScore (CountScore other, Annotation newAnnotation, double newCount) |
|  | |
|  | CountScore (AnnotationCollection<?extends Annotation > model, Annotation annotation) |
|  | |
|  | CountScore (AnnotationCollection<?extends Annotation > model, Annotation annotation, double regionTotal) |
|  | |
|  | CountScore (AnnotationCollection<?extends Annotation > model, Annotation annotation, boolean fullyContained) |
|  | |
|  | CountScore (AnnotationCollection<?extends Annotation > model, Annotation annotation, double regionTotal, boolean fullyContained) |
|  | |
|  | CountScore (Annotation annotation, double count, double regionTotal, double total) |
|  | |
| double | getCount () |
|  | |
| double | getScore () |
|  | |
| double | getTotal () |
|  | |
| double | getRegionTotal () |
|  | |
| double | getRPKM () |
|  | |
| double | getRPK () |
|  | |
| void | setCount (double count) |
|  | |
| void | setTotal (double total) |
|  | |
| void | setRegionTotal (double regionTotal) |
|  | |
| String | toString () |
|  | |
| boolean | equals (Object o) |
|  | |
| int | compareTo (CountScore o) |
|  | |
| int | hashCode () |
|  | |
| void | refreshPvalue () |
|  | |
| double | getPvalue () |
|  | |
| Public Member Functions inherited from umms.core.model.score.WindowScore.AbstractWindowScore | |
|  | AbstractWindowScore (Annotation t) |
|  | |
| Annotation | getAnnotation () |
|  | |

|  |  |
| --- | --- |
| Static Public Attributes | |
| static double | RPKM\_OFFSET = 0.001 |
|  | |
| Static Public Attributes inherited from umms.core.model.score.CountScore | |
| static double | DEFAULT\_REGION\_TOTAL = -1.0 |
|  | |

|  |  |
| --- | --- |
| Additional Inherited Members | |
| Static Public Member Functions inherited from umms.core.model.score.CountScore | |
| static double | asRPKM (double count, double total, int windowSize) |
|  | |
| Protected Attributes inherited from umms.core.model.score.WindowScore.AbstractWindowScore | |
| Annotation | annotation |
|  | |

## Constructor & Destructor Documentation

|  |  |  |  |  |  |
| --- | --- | --- | --- | --- | --- |
| umms.core.model.score.RatioScore.RatioScore | ( | Annotation | *a* | ) |  |

|  |  |  |  |
| --- | --- | --- | --- |
| umms.core.model.score.RatioScore.RatioScore | ( | Annotation | *numerator*, |
|  |  | Annotation | *denominator*, |
|  |  | double | *nTotal*, |
|  |  | double | *dTotal* |
|  | ) |  |  |

|  |  |  |  |
| --- | --- | --- | --- |
| umms.core.model.score.RatioScore.RatioScore | ( | Annotation | *numerator*, |
|  |  | Annotation | *denominator*, |
|  |  | double | *nTotal*, |
|  |  | double | *dTotal*, |
|  |  | double | *nRegionTotal*, |
|  |  | double | *dRegionTotal* |
|  | ) |  |  |

Here is the call graph for this function:

|  |  |  |  |
| --- | --- | --- | --- |
| umms.core.model.score.RatioScore.RatioScore | ( | CountScore | *numerator*, |
|  |  | CountScore | *denominator* |
|  | ) |  |  |

Here is the call graph for this function:

|  |  |  |  |
| --- | --- | --- | --- |
| umms.core.model.score.RatioScore.RatioScore | ( | AnnotationCollection<?extends Annotation > | *numerator*, |
|  |  | AnnotationCollection<?extends Annotation > | *denominator*, |
|  |  | Annotation | *annotation* |
|  | ) |  |  |

Here is the call graph for this function:

|  |  |  |  |
| --- | --- | --- | --- |
| umms.core.model.score.RatioScore.RatioScore | ( | AnnotationCollection<?extends Annotation > | *numerator*, |
|  |  | AnnotationCollection<?extends Annotation > | *denominator*, |
|  |  | Annotation | *annotation*, |
|  |  | double | *numeratorRegionTotal*, |
|  |  | double | *denominatorRegionTotal* |
|  | ) |  |  |

Here is the call graph for this function:

## Member Function Documentation

|  |  |  |  |  |
| --- | --- | --- | --- | --- |
| double umms.core.model.score.RatioScore.getDenominatorCount | ( |  | ) |  |

Here is the caller graph for this function:

|  |  |  |  |  |
| --- | --- | --- | --- | --- |
| double umms.core.model.score.RatioScore.getDenominatorRegionTotal | ( |  | ) |  |

Here is the caller graph for this function:

|  |  |  |  |  |
| --- | --- | --- | --- | --- |
| double umms.core.model.score.RatioScore.getDenominatorRPKM | ( |  | ) |  |

Here is the call graph for this function:

Here is the caller graph for this function:

|  |  |  |  |  |
| --- | --- | --- | --- | --- |
| double umms.core.model.score.RatioScore.getDenominatorTotal | ( |  | ) |  |

Here is the caller graph for this function:

|  |  |  |  |  |
| --- | --- | --- | --- | --- |
| double umms.core.model.score.RatioScore.getDepletionBinomialPValue | ( |  | ) |  |

Binomial test: combine numerator and denominator counts, then calculate cumulative probability of seeing less than this proportion

Returns
:   p-value for the numerator having less reads than expected.

Here is the call graph for this function:

Here is the caller graph for this function:

|  |  |  |  |  |
| --- | --- | --- | --- | --- |
| double umms.core.model.score.RatioScore.getDepletionBinomialScore | ( |  | ) |  |

Binomial test: combine numerator and denominator counts, then calculate cumulative probability of seeing less than this proportion

Returns
:   -log10 p-value for the numerator having less reads than expected.

Here is the call graph for this function:

Here is the caller graph for this function:

|  |  |  |  |  |
| --- | --- | --- | --- | --- |
| double umms.core.model.score.RatioScore.getEnrichmentBinomialPValue | ( |  | ) |  |

Binomial test: combine numerator and denominator counts, then calculate cumulative probability of seeing >= this proportion

Returns
:   p-value for the numerator having more reads than expected

Here is the call graph for this function:

Here is the caller graph for this function:

|  |  |  |  |  |
| --- | --- | --- | --- | --- |
| double umms.core.model.score.RatioScore.getEnrichmentBinomialScore | ( |  | ) |  |

Binomial test: combine numerator and denominator counts, then calculate cumulative probability of seeing >= this proportion

Returns
:   -log10 p-value for the numerator having more reads than expected.

Here is the call graph for this function:

Here is the caller graph for this function:

|  |  |  |  |  |
| --- | --- | --- | --- | --- |
| double umms.core.model.score.RatioScore.getLog2Ratio | ( |  | ) |  |

Here is the call graph for this function:

Here is the caller graph for this function:

|  |  |  |  |  |
| --- | --- | --- | --- | --- |
| double umms.core.model.score.RatioScore.getLog2RegionRatio | ( |  | ) |  |

Here is the call graph for this function:

|  |  |  |  |  |
| --- | --- | --- | --- | --- |
| double umms.core.model.score.RatioScore.getNumeratorCount | ( |  | ) |  |

Here is the call graph for this function:

Here is the caller graph for this function:

|  |  |  |  |  |
| --- | --- | --- | --- | --- |
| double umms.core.model.score.RatioScore.getNumeratorRegionTotal | ( |  | ) |  |

Here is the call graph for this function:

Here is the caller graph for this function:

|  |  |  |  |  |
| --- | --- | --- | --- | --- |
| double umms.core.model.score.RatioScore.getNumeratorRPKM | ( |  | ) |  |

Here is the call graph for this function:

Here is the caller graph for this function:

|  |  |  |  |  |
| --- | --- | --- | --- | --- |
| double umms.core.model.score.RatioScore.getNumeratorTotal | ( |  | ) |  |

Here is the call graph for this function:

Here is the caller graph for this function:

|  |  |  |  |  |
| --- | --- | --- | --- | --- |
| double umms.core.model.score.RatioScore.getRatio | ( |  | ) |  |

Here is the call graph for this function:

Here is the caller graph for this function:

|  |  |  |  |  |
| --- | --- | --- | --- | --- |
| double umms.core.model.score.RatioScore.getRegionDenominatorRPKM | ( |  | ) |  |

Here is the call graph for this function:

Here is the caller graph for this function:

|  |  |  |  |  |
| --- | --- | --- | --- | --- |
| double umms.core.model.score.RatioScore.getRegionNumeratorRPKM | ( |  | ) |  |

Here is the call graph for this function:

Here is the caller graph for this function:

|  |  |  |  |  |
| --- | --- | --- | --- | --- |
| double umms.core.model.score.RatioScore.getRegionRatio | ( |  | ) |  |

Here is the call graph for this function:

Here is the caller graph for this function:

|  |  |  |  |  |
| --- | --- | --- | --- | --- |
| double umms.core.model.score.RatioScore.getScore | ( |  | ) |  |

Returns the number of reads overlapping this region

Returns
:   number of reds overlapping this window Returns the "score" as defined by each subclass (e.g. count, ratio, sum of annotation scores, etc.)

Implements umms.core.model.score.WindowScore.

Here is the call graph for this function:

Here is the caller graph for this function:

|  |  |  |  |  |  |
| --- | --- | --- | --- | --- | --- |
| void umms.core.model.score.RatioScore.setDenominatorCount | ( | double | *d* | ) |  |

Here is the caller graph for this function:

|  |  |  |  |  |  |
| --- | --- | --- | --- | --- | --- |
| void umms.core.model.score.RatioScore.setDenominatorRegionTotal | ( | double | *d* | ) |  |

Here is the caller graph for this function:

|  |  |  |  |  |  |
| --- | --- | --- | --- | --- | --- |
| void umms.core.model.score.RatioScore.setDenominatorTotal | ( | double | *d* | ) |  |

Here is the caller graph for this function:

|  |  |  |  |  |  |
| --- | --- | --- | --- | --- | --- |
| void umms.core.model.score.RatioScore.setNumeratorCount | ( | double | *d* | ) |  |

Here is the call graph for this function:

|  |  |  |  |  |  |
| --- | --- | --- | --- | --- | --- |
| void umms.core.model.score.RatioScore.setNumeratorRegionTotal | ( | double | *d* | ) |  |

Here is the call graph for this function:

Here is the caller graph for this function:

|  |  |  |  |  |  |
| --- | --- | --- | --- | --- | --- |
| void umms.core.model.score.RatioScore.setNumeratorTotal | ( | double | *d* | ) |  |

Here is the call graph for this function:

|  |  |  |  |  |
| --- | --- | --- | --- | --- |
| String umms.core.model.score.RatioScore.toString | ( |  | ) |  |

Here is the call graph for this function:

## Member Data Documentation

double umms.core.model.score.RatioScore.RPKM\_OFFSET = 0.001

static

---

The documentation for this class was generated from the following file:

- java/umms/core/model/score/RatioScore.java


---

Generated on Fri Aug 15 2014 15:11:13 for ESAT by  

 1.8.7
